# Workload and funding in general practice: a nationwide cross-sectional study in England

**DOI:** 10.3399/BJGP.2025.0564

**Published:** 2026-05-19

**Authors:** Laura Anselmi, Yiu-Shing Lau, Shaolin Wang, Michael Anderson, Evangelos Kontopantelis, Matt Sutton

**Affiliations:** 1 Division of Population Health, Health Services Research and Primary Care, University of Manchester, Manchester, UK; 2 Division of Informatics, Imaging and Data Sciences, University of Manchester, Manchester, UK; 3 Division of Family Medicine, Yong Loo Lin School of Medicine, National University of Singapore, Singapore, Singapore

**Keywords:** capitation, deprivation, financing, general practice, primary health care, workload

## Abstract

**Background:**

Funding based on population need is key to equitable health care. The formula for general practice capitation payments in England has not been updated for over 20 years and is based on crude workload weights.

**Aim:**

To estimate and describe the uplifts in practice payments required to bring them in line with updated and more precise needs-based workload weights.

**Design and setting:**

Cross-sectional study assessing the most updated routinely available data on 6213 non-atypical general practices in England with >1000 registered patients and complete information on payments as of 1 April 2024.

**Method:**

Updated workload weights were applied to publicly available practice data on patient age and gender, new registration, ethnicity, deprivation, and prevalence of 20 long-term conditions. The practice payment provided for input price variation was removed and the current workload payment per weighted patient for each practice and the funding required to uplift all practices to minimum thresholds were calculated.

**Results:**

Workload payment per patient was £92.66 on average, varying from £86.72 in the lowest to £99.91 in the highest deprivation decile. Workload payment per weighted patient varied from £81.40 (5th percentile) to £107.10 (95th percentile), and from £89.69 in the lowest to £96.40 in the highest deprivation decile. Uplifting payments to a minimum of £102.46 per weighted patient (90th percentile value) would increase total payments by 11.6% (£677.77 million per year) and payments per patient to £99.75 in the lowest and £108.18 in the highest deprivation decile.

**Conclusion:**

The workload element of the general practice funding formula should use updated weights. Additional concerns related to equity in outcomes and underfunding of practices in deprived areas should be addressed by adjustments or revisions of other elements of the funding formula.

## How this fits in

General practice capitation payments in England are based on an outdated formula, which is perceived to underfund practices in more deprived areas. Updated capitation weights, which are now available and favour practices in more deprived areas, should be applied. Uplifting all practices to a minimum payment threshold equivalent to the 90th percentile of current payments per weighted patient would require a total funding increase of approximately 12%. The underfunding of practices in deprived areas should be addressed by adjustments or revisions of other elements of the funding formula.

## Introduction

Ensuring a fair distribution of primary care funding is key for equitable and efficient health systems promoting universal access and supporting population health improvement.^1–4^ Resource allocation mechanisms based on funding formulae are used in many countries to distribute available funding for primary care according to need^5^ and should be updated frequently to reflect changing needs.^6^ However, funding formulae are often not updated to exploit better available data,^7,8^ partly because redistribution of funding may mean a loss of income for some providers and encounter resistance to change.^6^

England uses a mixed payment system for general practices. The weighted capitation com ponent covers about 50% of funding.^9,10^ It is informed by the Global Sum formula (also known as Carr-Hill), developed in 2004 and based on workload, with adjustments for additional needs, rurality, and geographical variations in input prices.^11^ There are longstanding concerns that this formula does not adequately reflect current needs, particularly those of practices in more deprived areas, and that current resources are not sufficient to support an increase in funding for more disadvantaged practices.^12,13^

In 2007, a formula review group recommended revising the measure of workload to capture appointment length and home visits, and to include morbidity indicators.^11,14^ In 2016, the Advisory Committee on Resource Allocation (ACRA) approved a formula for allocating funding for primary medical services that included deprivation in the workload model. This is used to fund local commissioners, but not for payments to practices.^15^ Despite these efforts, the formula used for practice payments has not been updated for over 20 years. The recent *Fit for the Future: 10 Year Health Plan for England* includes plans for a review of *‘how health need is reflected in nationally determined contracts, such* [as] *the Carr-Hill formula for general practice’*.^16^

A recent study has proposed a refined approach and calculated updated weights using nationally representative, patient-level data covering over 20% of patients and practices in England.^17^ Workload weights were generated by relating cost-weighted appointments with clinical professionals to a richer set of patient characteristics, using a needs-based utilisation approach similar to that used for other NHS funding formulae. Variations in activity across practices that are not attributable to measured patient characteristics, along with lower levels of activity for people from minority ethnic groups, have been set to zero to account partially for unmet needs. Patient characteristics included age and gender, new registration, ethnicity, deprivation, and morbidity, measured using 20 conditions recorded in primary care records. The inclusion of morbidity indicators in the workload model was shown to better represent the increased needs of patients in more deprived areas.

In the current study, these updated weights were applied to the most recent data on registered populations for all general practices in England and compared with the current distribution of payments. The additional funding required to increase payments per weighted patient to a minimum benchmark were then estimated.

## Method

### Data

The most updated routinely available data on 6213 non-atypical general practices in England with >1000 registered patients and complete information on payments, covering 62 895 502 patients at 1 April 2024, was used (Supplementary Box S1). The study used information on the number of patients:

- by age and sex at 1 April 2024;^18^
- by ethnic background (at 1 April 2018);^17,19^
- by deprivation decile, measured by the area of residence English Index of Multiple Deprivation (IMD) 2019, a small-area composite measure across seven relevant domains;^18,20^
- included in Quality and Outcomes Framework (QOF) disease registers for each condition in 2023/2024;^21^ and
- newly registered with the practice (between 1 July 2020 and 30 June 2021).^22^

Practice deprivation was computed as the weighted area-level deprivation score of registered patients, and practice rurality as the proportion of patients residing in rural areas.

Data on payments to general practices for Global Sum, primary medical services (PMS), and minimum practice income guarantee (MPIG),^23^ which are allocated based on the Global Sum formula, were used. The market forces factor (MFF) index was obtained from the 2023/2024 NHS allocations.^22^

### Analysis

The workload regression model is linear and the contribution of each characteristic, weighted by the updated model’s coefficients, is therefore additive.^17^ Accordingly, expected workload for each practice was calculated by the dot product of mean values of each patient characteristic (Supplementary Table S1 and S2, and Supplementary Box S1) and the coefficients from the workload model (see Supplementary Table S3).^17^ In line with the approach taken for other NHS formulae, the authors replaced the negative coefficients estimated for some ethnic groups with a value of zero because these are interpreted as reflecting unmet need.^17^ In this study, the authors also did not include in the computation of workload variations in activity between practices that are captured by the practice fixed effects as they may reflect differences in capacity.^17^ Each practice’s updated weighted patient was computed as the practice’s expected workload divided by the number of registered patients, and then standardised around the national mean value.

To isolate the workload from the cost element, the authors first removed £2.18 for each patient residing in the Greater London Authority area and then deflated practice Global Sum (including residual MPIG payments) and PMS payments by the MFF adjustment. The MFF is a factor ≥1 applied to 48% of payments, the share spent on salary, to adjust for local input prices.^24^ For all practices the authors divided the current payment by 0.52 + 0.48 * MFF. The authors could not remove the rurality adjustment. For practices receiving Global Sum payments, each practice’s weighted patient was computed by dividing payment (total Global Sum payment) and workload payment (Global Sum payment net of cost adjustments) by patient list size^18^ and standardising around the mean value. The Carr-Hill weighted patient was also computed by dividing number of weighted patients by the number of registered patients provided alongside practice payments data.^23^

First, the authors compared the distributions of the updated weighted patients with the Global Sum and Carr-Hill weighted patients, and with the most recent weighted patients from the current primary medical care^22^ and prescribing^25^ NHS formulae. Second, variation in workload payments per weighted patient were summarised across all practices and by practice deprivation and rurality.

The authors then considered the approach taken when the Carr-Hill formula was first introduced and with current NHS pace-of-change policy, which progressively increases funding in places further below their needs-based targets. Workload payments per weighted patient were benchmarked to levels in better-funded practices, defined as practices at the 50th, 75th, or 90th percentile of the distribution. For each of these three scenarios, the additional funding required to uplift payments in all practices below the threshold was computed. Current and uplifted payments were examined across all practices and by practice deprivation and rurality. Results for integrated care boards (ICBs, as defined at 1 April 2024), the organisations in charge of commissioning local health services, are also shown.

## Results

Around a standardised average value of one, workload weight per patient varied between 0.815 (5th percentile) and 1.158 (95th percentile) at practice level, between 0.973 on average for the least deprived and 1.038 for the most deprived decile, and between 0.949 for the least rural and 1.061 for the most rural quintile (Table 1 and Supplementary Table S4).

**Table 1.** Distribution of average weighted patient across practices^a^

Category	Practices, *n*	Registered patients, *n*	Deprivation score, mean	Average weighted patient
**Overall distribution**				
Mean	—	—	—	1
p5	—	—	—	0.815
p10	—	—	—	0.867
p25	—	—	—	0.937
p50	—	—	—	1.007
p75	—	—	—	1.071
p90	—	—	—	1.123
p95	—	—	—	1.158
p95–p5 gap	—	—	—	0.343
p95–p5 ratio	—	—	—	1.421:1
**Practice deprivation decile**				
1 (least deprived)	627	7 045 048	8.2	0.973
2	629	6 758 007	11.5	0.994
3	624	6 704 093	14.1	1.000
4	624	6 361 572	16.8	1.002
5	620	6 393 942	19.8	1.006
6	623	6 908 711	23.1	0.984
7	617	6 314 282	26.5	0.995
8	616	6 243 484	30.6	0.997
9	617	5 491 534	36.0	1.030
10 (most deprived)	616	4 674 829	46.7	1.038
Most–least gap	—	—	—	0.065
Most–least ratio	—	—	—	1.067:1
**Practice rurality quintile (proportion of patients in rural areas)**				
1 (0.00–0.0002)	1219	11 450 115	27.0	0.949
2 (>0.0002–0.003)	1253	11 730 632	27.0	0.967
3 (>0.003–0.03)	1250	12 825 355	24.3	0.992
4 (>0.03–0.25)	1241	14 942 329	18.8	1.022
5 (>0.25–1)	1250	11 947 071	14.9	1.061
Most–least gap	—	—	—	0.111
Most–least ratio	—	—	—	1.117:1

^a^Weights for practice list size are applied. p = percentile.

The total payment for Global Sum and PMS for practices in the current study’s dataset in 2023/2024 was £6313.72 million (£100.38 per patient), representing approximately 60% of payments made to practices under all schemes (£10 531.29 million in total and £167.44 per patient). After removing the payments made for the London adjustment and the MFF, Global Sum and PMS workload payments were £5827.99 million (£92.66 per patient), varying between £86.72 in the least and £99.91 in the most deprived decile, and between £87.02 in the least and £101.81 in the most rural quintile per patient (Supplementary Table S5).

**Figure 1.**
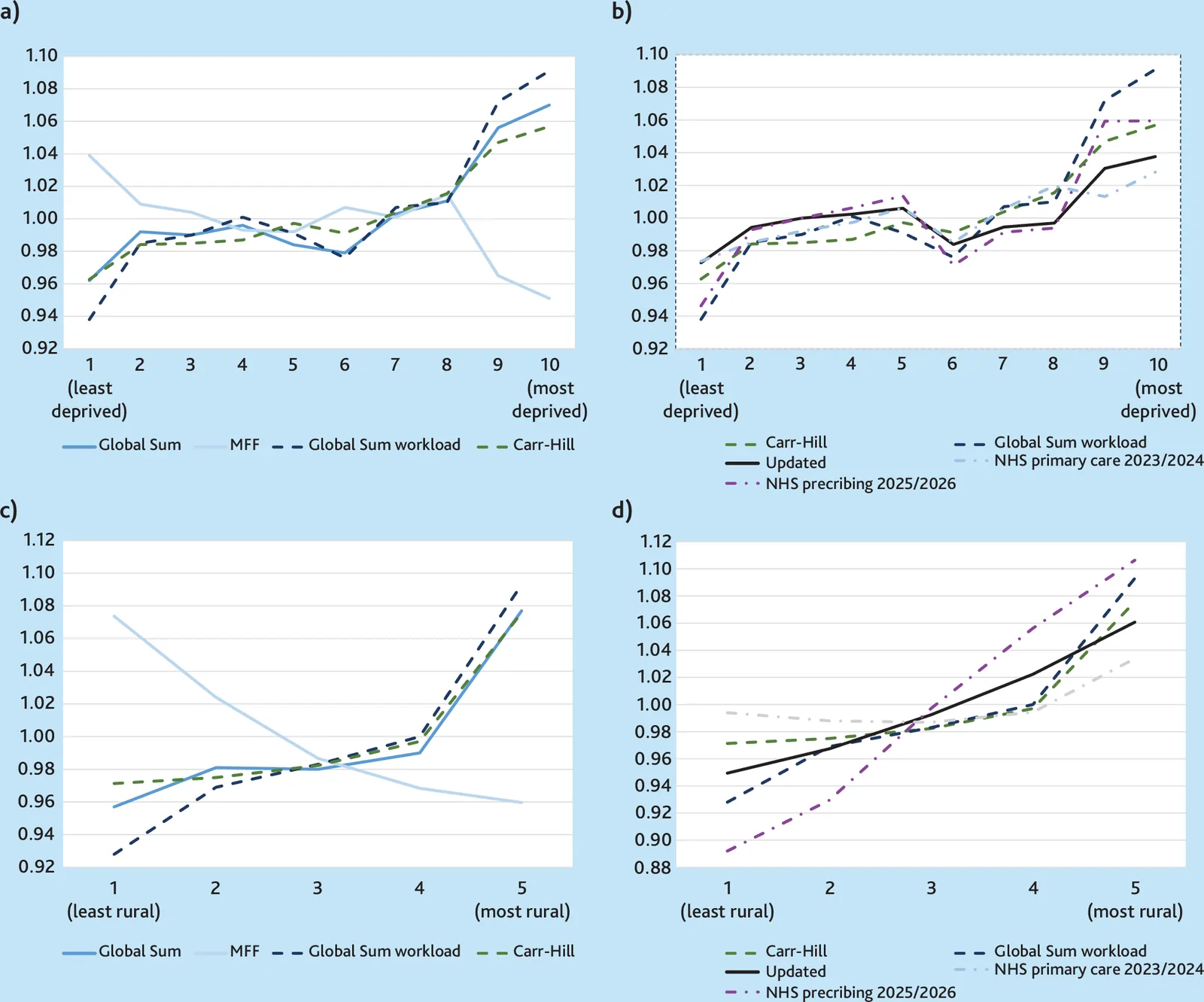
Comparisons of weighted patient: **a**) Carr-Hill, Global Sum (total), Global Sum workload (net of cost adjustments), MFF (market forces factor), by deprivation;** b**) Updated formula, Carr-Hill, Global Sum workload, NHS primary care formula, and NHS prescribing formula, by deprivation; **c**) Carr-Hill, Global Sum (total), Global Sum workload (net of cost adjustments), MFF (market forces factor), by rurality; and d) Updated formula, Carr-Hill, Global Sum workload, NHS primary care formula, and NHS prescribing formula, by rurality.

The Carr-Hill weighted patient were higher for practices serving populations in more deprived areas. The pattern accentuated for workload payments after removing the MFF factor (Figure 1a and Supplementary Table S6). The updated weights were less favourable to more deprived areas compared with Carr-Hill and were mostly in between the NHS primary care and NHS prescribing indices (Figure 1b and Supplementary Table S6). The patterns were similar when comparing weighted patients by rurality (Figure 1c and 1d, and Supplementary Table S6).

Current Global Sum and PMS workload payments per weighted patient varied between £81.40 and £107.10 at the 5th and 95th percentile (£83.57 at 10th, £92.11 at 50th, £97.14 at 75th, and £102.46 at 90th percentile), and between £89.69 in the least and £96.40 in the most deprived deciles (Supplementary Table S7). Comparisons with total payments per weighted patients are available in Supplementary Table S8.

Uplifting workload payments to all practices up to a minimum benchmark of £102.46 per weighted patient (90th percentile) would require a total of £6505.76 million (£ 103.44 per patient). This would involve a budget increase of £677.77 million per year (£10.78 per patient), which represents 11.6% of current workload payments. Payments per patient would increase to £99.75 in the lowest and £108.18 in the highest deprivation decile. Smaller increases of £391.82 million (£6.23 per patient) and of £184.73 million (£2.94 per patient) would be required to support uplifts to the 75th and 50th percentiles benchmarks, respectively (Table 2). Payments would increase in all deprivation deciles and rurality quintiles (Supplementary Figure S1) and for all ICBs (Supplementary Figure S2 and Supplementary Table S9).

**Table 2.** Current and projected workload payments under different uplift scenarios^a,b^

Category	Current, £	Uplift to 50th p, £		Uplift to 75th p, £		Uplift to 90th p, £	
		Level	Increase	Level	Increase	Level	Increase
**Total (000 000s**)	5827.99	6012.72	184.73	6219.81	391.82	6505.76	677.77
**Per patient**	92.66	95.60	2.94	98.89	6.23	103.44	10.78
**Overall distribution**							
p5	72.74	77.86	0.00	81.07	0.00	84.93	0.00
p10	76.67	81.99	0.00	85.48	0.00	89.81	0.67
p25	83.48	87.73	0.00	91.76	0.63	96.48	5.94
p50	91.46	94.74	0.49	98.74	5.57	103.57	10.88
p75	100.99	102.28	4.89	105.32	9.85	110.28	15.16
p90	108.98	109.54	8.46	111.78	13.52	116.19	18.89
p95	113.89	114.39	10.75	115.85	15.85	120.09	21.36
p95–p5 gap	41.15	36.53	10.75	34.78	15.85	35.16	21.36
p95–p5 ratio	1.6:1	1.5:1	—	1.4:1	—	1.4:1	—
**Practice deprivation decile**							
1 (least deprived)	86.72	90.73	4.00	94.82	8.09	99.75	13.03
2	90.19	94.01	3.82	97.77	7.58	102.58	12.38
3	90.69	94.11	3.42	97.85	7.17	102.69	12.00
4	91.70	94.84	3.14	98.44	6.74	103.24	11.53
5	92.09	95.38	3.29	98.82	6.73	103.50	11.41
6	91.85	94.32	2.47	97.41	5.56	101.83	9.98
7	93.37	96.12	2.75	99.23	5.86	103.57	10.21
8	94.45	96.63	2.18	99.40	4.95	103.77	9.32
9	99.51	100.99	1.48	103.54	4.03	107.75	8.24
10 (most deprived)	99.91	102.17	2.26	104.45	4.54	108.18	8.27
Most–least gap	13.19	11.45	1.74	9.63	3.56	8.43	4.76
Most–least ratio	1.2:1	1.1:1	—	1.1:1	—	1.1:1	—
**Practice rurality quintile (proportion of patients in rural areas)**							
1 (0.00–0.0002)	87.02	90.34	3.32	93.68	6.67	98.08	11.07
2 (>0.0002–0.003)	89.75	93.07	3.32	96.40	6.65	100.77	11.02
3 (>0.003–0.03)	91.17	94.57	3.40	98.02	6.84	102.70	11.52
4 (>0.03–0.25)	93.24	96.52	3.28	100.26	7.02	105.17	11.93
5 (>0.25–1)	101.81	103.06	1.26	105.56	3.75	109.82	8.02
Most–least gap	14.79	12.72	2.07	11.87	2.92	11.74	3.05
Most–least ratio	1.2:1	1.1:1	—	1.1:1	—	1.1:1	—

^a^Workload payments include Global Sum, primary medical services (PMS), and minimum practice income guarantee payments for workload, after removing London and market forces factor adjustment. Weights for practice list size are applied. Thresholds for uplift: £92.11 (50th percentile); £97.14 (75th percentile); and £102.46 (90th percentile) (Supplementary Table S7). ^b^Values may differ from expected total becasue of rounding. p = percentile.

## Discussion

### Summary

The distribution of current payments to general practices in England were compared with updated needs-based capitation weights^17^ reflecting expected workload differentiated by patient age, gender, ethnicity, new registration, area-level deprivation, and morbidity. Average workload payment per patient per year was £92.66. This varied substantially between practices, from £86.72 in the least to £99.91 in the most deprived decile. The payment per weighted patient varied from £81.40 (5th percentile) to £107.10 (95th percentile), and between £89.69 in the least and £96.40 in the most deprived deciles. Compared with Carr-Hill, the differences in average practice weighted patients by deprivation were narrower in the updated weights.

Uplifting workload payments to all practices up to a minimum benchmark of £102.46 per weighted patients (90th percentile) would require an increase of £677.77 million (£10.78 per patient), equivalent to 11.6%. Smaller increases of £391.82 million (£6.23 per patient) and £184.73 million (£2.94 per patient) would be required to uplift to the 75th and 50th percentiles, respectively. Uplifting the current funding to the three alternative benchmarks would maintain higher payments per patient in the most deprived and rural practices. However, the ratio between payments in most to least deprived practices would slightly reduce.

### Strengths and limitations

The Global Sum (Carr-Hill) formula was based on data from 240 general practices (3% of the 8486 total) in 2004. Workload was differentiated by age, sex, and new registrations, and was quantified as average file opening times for surgery-based appointments, rescaled by the salary of the consulting professional. Appointment length for home visits and residential and nursing homes was adjusted using information from separate studies. An area-level adjustment for additional needs, based on standardised rates of limiting longstanding illness and <65 years mortality, and estimated on responses to the Health Survey for England 1998–2000, was also applied.^11^

The new weights are based on updated and more representative data, and on a more refined measure of workload. Workload reflects appointments with GPs, nurses, healthcare assistants, and other clinical staff, differentiated not only by age and gender, but also by ethnicity, deprivation, and morbidity.^17^ The model estimating workload included controls for differences in supply to ensure that the patient weights did not reflect differences in access. This needs-based utilisation approach aligns with the most advanced person-based formulae in use for NHS allocations, regularly reviewed and updated by the ACRA.^26–28^

The inclusion of morbidity in the workload formula increases needs discrimination between practices and the estimated workload in more deprived practices (Supplementary Table S4).^14,17^ The current study was constrained by information routinely available at practice level. In this study, the authors could only use 20 conditions diagnosed in primary care and assume independent and additive effects of patient characteristics. The inclusion of additional diagnostic information and interactions between patient characteristics in the workload model did not substantially affect practice weighted patient.^17^ However, data should be made available to include richer sets of conditions recorded in primary or secondary care to account for additional cases and needs currently unmet in primary care.^29^

Although the updated weights adjust to some extent for unmet needs, by setting to zero negative coefficients on certain ethnic groups and adjusting for practice variations in activity that are more likely attributable to capacity rather than patient characteristics, they may still not fully reflect the needs of diverse populations.^17^ The updated workload weights do not account for appointment content and duration, which may vary with patients’ capacity to engage and socioeconomic background, and for which there are currently no available measures.^17^ The weights in the current study therefore only reflect clinical workload and may not fully reflect social needs and the evolving role of general practice, including extended scope of services and the work of new staff roles.^30^

### Comparison with existing literature

England has a long history of using funding formulae refreshed periodically to allocate health budgets to local organisations in charge of commissioning services.^6,31^ Formulae provide measures of relative need for different services, estimated as expected utilisation based on need characteristics after factoring out differences in supply and adjusting for underutilisation attributable to unmet needs.^32,33^ There are further separate adjustments for unmet needs and inequalities, as well as differences in costs of service delivery.^26^ The current study has demonstrated the applicability of recently developed updated needs-based workload weights derived using the NHS approach to funding formulae for general practice to inform payments. The distribution of the updated needs-based workload weighted patients is aligned with the most recent NHS need indices for primary medical care and for prescribing.^15,34^

### Implications for research and practice

Updated weights reflecting deprivation and morbidity while adjusting for differences in access should be applied. According to the current study’s analysis, the workload component of the Carr-Hill formula places higher weights on deprived areas compared with the updated weights. This is because of the sizeable weightings for two area-level indicators of premature longstanding illness and mortality. The updated weights offer a more precise measure of relative expected workload by directly reflecting differences in needs associated with a wider range of patient characteristics. Patients residing in more deprived areas and with diagnosed conditions are attributed higher weights, but the adjustments are not as large as in the original Carr-Hill formula. Further adjustments for inequalities could be updated and developed separately in line with the approach in other NHS formulae,^26^ or developed through advanced methods applied to improved data further discriminating workload between patients and areas. In addition, other elements of the formula, such as the current MFF adjustment for input prices, which redirects resources toward less deprived practices, should be re-evaluated to respond to the higher needs of practices in more deprived and rural areas.

There is evidence that GPs operating in areas of higher deprivation face additional pressures and workload,^35^ reporting more pressures from patients with complex needs, insufficient resources, and difficulty finding locums.^36^ Further research should focus on understanding and quantifying the effects of deprivation on workload beyond area-level characteristics and increased morbidity.

In line with the NHS pace-of-change policy, the implementation of any adjustment to the current distributions of funding may be contingent on ensuring no reduction in payments for any practice, unless the government is prepared to increase funding for general practice. Although increasing the total amount of resources for general practice may be fiscally challenging, the updated weights should be applied by uplifting practices to an agreed minimum level of funding per weighted patient, including in the most deprived and most rural and remote areas.

An increase of £184.73 million (£2.94 per patient) and £677.77 million (£10.78 per patient) would be required for uplifting to the level of the 50th and 90th percentile of payments per weighted patients, respectively, and to align payments to the new weights.

In the longer term, the implementation of needs-based capitation weights that reflect differences in relative expected workload across practices in England will require routinely updated person-level primary care records linked with practice and area characteristics. Such data should be made available to facilitate improvements in funding formulae for general practice that better characterise the needs of diverse populations.

The current formula is outdated, and updated needs-based workload weights should be used to inform payments to general practices. Additional concerns related to equity in outcomes and underfunding of practices in deprived areas should be addressed separately. They could be addressed through specific adjustments, by revising other elements of the funding formula, or by considering redistributions of payments in schemes that directly target the challenges faced by those practices.

## Data

All data used are referenced and accessible.
